# Root Patterning: Tuning SHORT ROOT Function Creates Diversity in Form

**DOI:** 10.3389/fpls.2021.745861

**Published:** 2021-09-30

**Authors:** Marcela Hernández-Coronado, Carlos Ortiz-Ramírez

**Affiliations:** UGA Laboratorio Nacional de Genómica para la Biodiversidad, CINVESTAV Irapuato, Guanajuato, Mexico

**Keywords:** root patterning, arabidopsis, short root, monocots, morphological diversity, root development, nodulation

## Abstract

Roots have a fundamental role in plant growth and adaptation to different environments. Diversity in root morphology and architecture enables plants to acquire water and nutrients in contrasting substrate conditions, resist biotic and abiotic stress, and develop symbiotic associations. At its most fundamental level, morphology is determined by discrete changes in tissue patterning. Differences in the number and arrangement of the cell layers in the root can change tissue structure, as well as root length and girth, affecting important productivity traits. Therefore, understanding the molecular mechanisms controlling variation in developmental patterning is an important goal in biology. The ground tissue (GT) system is an ideal model to study the genetic basis of morphological diversity because it displays great interspecific variability in cell layer number. In addition, the genetic circuit controlling GT patterning in *Arabidopsis thaliana* has been well described, although little is known about species with more complex root anatomies. In this review, we will describe the *Arabidopsis* model for root radial patterning and present recent progress in elucidating the genetic circuitry controlling GT patterning in monocots and the legume *Medicago truncatula (Mt)*, species that develop roots with more complex anatomies and multilayered cortex.

## Introduction

Root morphological diversity can be studied at different scales: macroscopically (i.e., at the level of root architecture, length, branching angle, and number of secondary roots) and microscopically, as changes in tissue pattering, like the number and arrangement of cell layers. Although root system architecture has been studied extensively, the relationship between patterning and root phenotypic diversity and adaptation has seldom been explored. Recently, it was proposed that root morphological trait variation across species and across biomes is most strongly influenced by root diameter, a character that depends on tissue layer number and organization (Ma et al., 2018).

Roots consist of three fundamental tissue types arranged radially as concentric layers: the epidermis on the outside; the ground tissue at the middle, consisting of one layer of endodermis and one to many layers of cortex; and a core of vascular elements plus pericycle called the stele (Dolan et al., 1993). Differences in the number and/or arrangement of these cell layers are not only relevant to explain diversity in root form, but they also give rise to important functional traits that have an impact on plant fitness and productivity (Lux et al., 2004). For example, plants living in waterlogged soils, such as rice, develop air filled cells from the cortex called aerenchyma (Yamauchi et al., 2018). Desert plants can accumulate water in their roots thanks to the proliferation of storage parenchyma from cortical cells, and a similar process allows plants to save starch to overwinter in difficult weather (Fahn, 1990; Lux and Inanaga, 1997). Halophyte plants growing in soils with toxic salt levels develop two suberized endodermis layers instead of one, which helps to isolate the root vasculature from the apoplast that is in direct contact with the soil (Inan et al., 2004).

At the basis of these developmental adaptations is the activity of genetic circuits that coordinate cell division activity and cell identity at the root apical meristem (RAM). Here, a set of stem cells, called initials, divide asymmetrically to produce daughter cells that give rise to epidermal, ground tissue, and vascular lineages (Esau, 1977; Benfey et al., 1993). While anticlinal divisions of initial cells will contribute to root length, periclinal divisions generate extra cell layers along the radial axis increasing girth. For example, in *Arabidopsis* a periclinal division of the cortex/endodermal initial gives rise to a single endodermis and a single cortex cell layer (Scheres et al., 1996). However, in species with roots that have multiple cortical layers the cortex/endodermal initial may undergo several divisions early in development producing thicker roots (Heimsch and Seago, 2008). There is limited information on the regulation of genetic circuits that produce different morphologies between closely related plant species, and even between roots from the same plant. Therefore, comparative intra- and interspecific developmental studies between roots with different morphologies are important for pinpointing the genetic basis of variation.

In this context, the ground tissue system is an ideal model to make such comparative studies since (1) it shows great variation in patterning, not only between closely related species, but also between root types from the same plant. e.g., rice roots can have between two to eleven ground tissue layers, depending on the root type (Clark and Harris, 1981). Maize develops between 8 and 15 layers (Bennetzen and Hake, 2009). (2) In many species, the ground tissue forms the bulk of the root; therefore, variations in the number of cortical cell layers have a significant effect on root form. (3) The genetic circuit controlling GT patterning in *Arabidopsis* is well described and serves as a model to search for modifications in the activity of homologous genes in species with more elaborated anatomies.

In this review, we will first describe the genetic circuit that controls development of the GT in *Arabidopsis*. Then, we will discuss the latest research in species with different anatomies and multilayered cortex. Finally, we will contrast the classical and new emergent models of root radial patterning and how they can be applied to explain morphological diversity.

## Differences in GT Development

**Figure 1:**
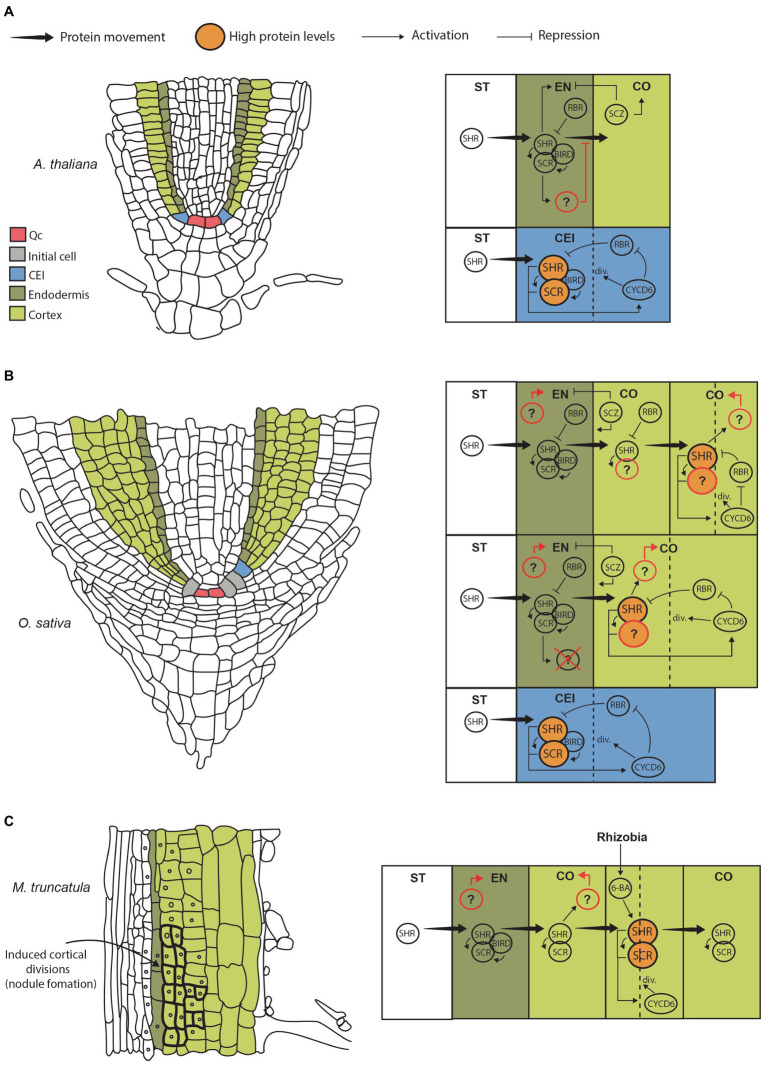
Genetic models for GT formation and cortex expansion in different species. **(A)** Cartoon showing a longitudinal section from an Arabidopsis thaliana root highlighting initial and GT cells (left). Diagram representing the CEI, endodermis, cortex and stele, where the main genetic pathways contributing to GT formation are depicted (right). Briefly, SHR protein moves from the stele into the CEI where it induces SCR expression. SHR and SCR form a complex that moves FIGURE 1  | into the nucleus and activates CYCD6;1, which in turns prevents the negative regulator RBR from interacting with the SHR/SCR complex. This results in high levels of SHR (big circles), which trigger asymmetric cell division giving rise to cortex and endodermis. Cell division resets the circuit from its on state. Presumably, SHR/SCR complex levels are maintained low by RBR preventing further divisions. SCZ helps in establishing tissue boundaries by inducing cortical identity and repressing endodermal identity. Importantly, SHR protein movement is limited to the endodermis either directly by biding to SCR and BIRD proteins, or indirectly by a SCR-induced factor that has been hypothesized to limit its movement (Wu et al., 2014; question mark). **(B)** Cartoon of a longitudinal sectional from rice root (left). Diagram representing the putative pathways contributing to GT formation and multilayered cortex in monocots (right). Note that SHR movement is not restricted to the endodermis; hence, the factor limiting its movement in Arabidopsis is either not present or has modified activity. We propose that SHR moves into the cortex where it can accumulate at high levels (big circles) activating and flipping the SHR/SCR/CYCLIND6;1/RBR circuit to its “on” state. This movement and activation are maintained until several cortical layers are produced. This model also assumes that SHR does not induce endodermal differentiation. Therefore, the factors that specify endodermal and cortical cell fate in monocots are unknown (question marks). It is also unclear if SHR interacts with SCR in the cortex as SCR protein localization in rice and other monocots is unknown. **(C)** Cartoon of a longitudinal sectional from Medicago root differentiation zone (left). Diagram representing the putative pathway contributing to cortex formative divisions giving rise to the nodule primordium (right). This model assumes that SHR and SCR proteins are present and interact throughout the root cortex. This allows the expression of the SHR/SCR/CYCLIND6;1/RBR circuit in cortical cells; however, the circuit is kept in an “off” state in the absence of rhizobia (low SHR levels). When symbiont signals are perceived by individual cells, 6-BA triggers local SHR accumulation in those domains flipping the circuit to its “on” state and triggering divisions.

Root tissue systems originate in the RAM, a region located near the tip of the root where cells remain undifferentiated. Here, a set of stem cell populations called initial cells undergo asymmetric cell division to produce different lineages. In *Arabidopsis*, there is four types of initials or stem cells: a population giving rise to vascular tissues, a population generating columella cells, a common initial producing the ground tissue (endodermis and cortex), and a common initial that originates root cap and epidermis (Pauluzzi et al., 2012). In the case of the ground tissue, the cortex/endodermis initials (CEIs) regenerate themselves by an anticlinal cell division, giving rise to the cortex endodermis initial daughter cell (CEID; Figures 1A,B). The CEID then undergoes one periclinal division to form two tissue layers: endodermis and cortex (Benfey et al., 1993). Thus, in primary development the *Arabidopsis* root has a single endodermal and a single cortex layer. Later in development an extra layer of cortex, called the middle cortex (MC), originates from the endodermis by an asymmetric periclinal division (Baum et al., 2002; Paquette and Benfey, 2005).

Important differences exist between root development in *Arabidopsis* and monocots, like rice and maize. In monocots, the epidermis and the ground tissue originate from the same initial cell. Therefore, a first asymmetrical division generates the CEI and the epidermis. Subsequently, the CEI will divide further giving rise to endodermis and cortex (Di Ruocco et al., 2018b; Figure 1B, left). Another important difference in the ability of most monocot roots to produce a multilayered cortex. It has been proposed that formation of several cortex layers is achieved by sustained periclinal cell divisions either of a single CEI or by divisions of multiple independent CEIs (Pauluzzi et al., 2012). In rice, there is a large variation in the number cortex cell layers depending on the root type. Small lateral roots have no cortex, while large laterals have 3; radical roots have 5, and crown roots have 10 (Clark and Harris, 1981). It is likely that precise modulation of the genetic circuit that controls CEI periclinal divisions underlies cortex layer number variation in rice, and this may be representative of a mechanism giving rise to interspecific ground tissue variation. Importantly, root diameter among rice root types is closely correlated with cortex cell layer number (Henry et al., 2015). Therefore, modulation of the CEI division program affects root size and function. Although this diversity in cortex development is most evident in monocots, it is not unique to this group. Some closely related dicot species also present differences in cortex layer number. For example, *Cardamine hirsuta,* a close *Arabidopsis* relative, has two cortex layers instead of one (Di Ruocco et al., 2018a). The wild tomato species *Solanum pennellii* has two cortex layers, while the domesticated *Solanum lycopersicum* has three, suggesting that differences in the genetic network controlling CEI divisions can evolve rapidly (Ron et al., 2013).

There is increasing evidence that the SHORT ROOT (*SHR*)/SCARECROW (*SCR*) genetic pathway, initially described in *Arabidopsis*, also controls GT formation across species and that modifications in the activity of this pathway originate a multilayered cortex.

## The SHR/SCR Pathway Controls GT Formative Divisions

As mentioned before, the asymmetric division of the CEI is essential for GT formation. Genetically, this process is controlled by the activity of *SHR* and *SCR* transcription factors (TFs; Helariutta et al., 2000). These genes are members of the GRAS protein domain family, and together they activate the genetic pathway that determines radial patterning. Mechanistically, *SHR* functions as a mobile signal that regulates cell division and cell fate specification. *SHR* transcript is expressed in the stele; however, the protein moves intercellularly through plasmodesmata into the adjacent CEI, CEID, and endodermis layer where it induces the expression of the downstream target *SCR* (Nakajima et al., 2001). SHR and SCR then form a complex at the protein level that moves into the nucleus of the CEI inducing the expression of a D-type cyclin, *CYCLIND6;1* (*CYCD6;1*) in *Arabidopsis*. This triggers cell division allowing the differentiation of the clonally related cortex and endodermal cell layers (Sozzani et al., 2010; Figure 1A, right).

Importantly, this division of the CEI depends on a bistable circuit that functions as an on/off switch. Circuit stability is determined by *CYCD6;1* activity and the cell cycle regulator RETINOBLASTOMA-RELATED protein (*RBR*). Interaction of RBR with SCR reduces SHR/SCR complex formation, therefore decreasing SHR, SCR, and CYCD6;1 levels in a negative feedback loop that prevents cell division (“off” state; Cruz-Ramirez et al., 2012). At the same time, a positive feedback loop is encoded in this circuit because CYCD6;1 can negatively regulate RBR through phosphorylation (Figure 1A, right). Thus, increasing *CYCD6;1* activity shifts back the circuit to an “on” state. Simulations demonstrate that high levels of SHR influx into the CEI are needed to activate sufficient *CYCD6;1* and gain the potential for asymmetric cell division. Moreover, very high levels ensure flipping to the “on” state triggering division. Finally, the bistable switch is reset when the cell undergoes division and proteins are degraded (Cruz-Ramirez et al., 2012).

Because SHR levels determine the potential of GT initial cells to undergo asymmetric divisions, the movement and magnitude of SHR fluxes into adjacent tissues have to be tightly regulated during GT formation. A prediction that can be extrapolated from current models is that further movement into the adjacent cell layers or GT initials will cause further activation of *SCR* and *CYCD6;1* and thus cell division, increasing GT layer number. In *Arabidopsis,* it has been shown that SCR itself can regulate SHR movement by binding and sequestering SHR into the nucleus, which effectively limits the amount of protein that can move into the next layer (Heidstra et al., 2004; Cui et al., 2007). Therefore, SHR limits its own movement by inducing *SCR* expression forming another feedback loop. This has been demonstrated by the reduction of *SCR* expression *via* RNAi, which results in increased SHR mobility and production of more GT layers (Cui et al., 2007). In addition, the *SHR/SCR* pathway induces the expression of the *BIRD* zinc finger proteins JACKDAW (*JKD*), MAGPIE (*MGP*), BALDIBIS, and NUTCRACKER that together with SCR also reduce SHR mobility and contribute to maintain tissue boundaries (Welch et al., 2007; Long et al., 2017).

After CEI division, the daughter cells acquire either endodermis or cortex identity. *SHR* also has an essential role in this process since experiments in *Arabidopsis* show it is necessary for endodermis cell fate specification. *AtSHR* loss-of-function roots lack an endodermis and display a single GT layer with cortex identity. On the other hand, *SCR* mutants also display a single GT layer, but this has a mix of endodermal and cortex markers. Importantly, double mutants of *SCR* and its close homolog *SCARECROW-LIKE23* lack an endodermis similarly to SHR mutant, indicating that these TFs act together with *SHR* to specify cell identity. (Benfey et al., 1993; van den Berg et al., 1995; Long et al., 2015). Another transcription factor that is important for defining GT cell fate during formative divisions is SCHIZORIZA (*SCZ*), a member of the heat-shock TF family. SCZ is important for cortex fate specification, since loss-of-function mutants develop two ground tissue layers both with endodermis identity. Conversely, *SCZ* overexpression from the 35s promoter results in ectopic cell divisions in epidermal and lateral root cap tissues in which high expression of the cortex marker Co2 is detected (ten Hove et al., 2010). Furthermore, *SCZ* has role in regulating correct cell fate separation and establishment of tissue boundaries across the root. Mutants show extra layers in ground, epidermal and lateral root cap tissues that express a mix of cell identity makers (Pernas et al., 2010).

## Middle Cortex Formation

In addition to activating cell division and specifying cell identity, *SHR* has a role in post-embryonic development of cortical tissue. In *Arabidopsis* roots, an additional cortex layer, called the middle cortex (MC), develops approximately 10–14days after germination. This layer does not originate from the CEI as described previously, but from an asynchronous division of the endodermis that generates one extra cell layer that rapidly acquires cortex identity (Paquette and Benfey, 2005). *SHR* and *PHABULOSA* trigger this formative division through the reactivation of *CYCD6;1* (Sozzani et al., 2010; Bertolotti et al., 2021). However, the timing of this division is important and is regulated by the hormone gibberellic acid (GA). High levels of GA inhibit MC formation early after germination in a *SHR*-dependent process (Paquette and Benfey, 2005; Cui and Benfey, 2009). Surprisingly, *SCR* acts antagonistically to *SHR* by repressing MC formation, as loss-of-function mutants initiate MC prematurely (Paquette and Benfey, 2005). Notably, the *SHR*, *SCR,* and *GA* pathways converge on the regulation of the transcription factor *SCARECROW LIKE 3*. This TF induces GA activity and itself is positively regulated by *SHR* and *SCR* forming a feedback loop that contributes to time MC formation (Heo et al., 2011; Koizumi et al., 2012; Gong et al., 2016).

## SHR in the Development of Multilayered Cortex

In contrast to what has been reported in *Arabidopsis*, some functional aspects of *SHR* activity, such as mobility and fate specification, appear not to be conserved in other species. In recent years, compelling evidence has emerged for a role of *SHR* in the specification and control of cortex layer number. Most of this new evidence comes from the study of *SHR* homologs in monocots, which develop roots with a multilayered cortex (Rebouillat et al., 2008). Notably, it was found that two SHR homologues from rice (*Oryza sativa)* and one homolog from *Brachipodium distachyon* have increased intercellular movement compared to *Arabidopsis* SHR. When expressed heterologously in the stele of *Arabidopsis* roots, movement of the monocot SHR proteins was not limited to the adjacent endodermis but instead they moved 3 to 6 layers past the stele. All monocot SHR proteins were shown to strongly interact with *At*SCR, *At*JKD, and *At*MGP; hence, hypermobility could not be explained by a lack of interaction (and nuclear sequestration) between these transcription factors. The observed increase in SHR mobility was correlated with a similar increase in GT layer number. Therefore, SHR movement seems to be sufficient to activate the *SHR/SCR/CYCLIND6;1* circuit causing extra divisions and an enlarged GT (Wu et al., 2014; Figure 1B, right). Importantly, by performing a propidium iodide exclusion test and analyses of cell-type specific markers, Wu, et al. determined that the extra cell layers created by monocot SHR hypermobility had cortex identity. Hence, although monocot *SHR* homologs generate extra GT layers in *Arabidopsis*, just a single endodermis layer, the one in direct contact with the stele, was specified.

The fact that the experiments described before were performed in a heterologous system has both strengths and limitations. In this context, a recent study describing maize (*Zea mays*) endogenous *SHR* function provides valuable insight into root development. Both *ZmSHR* transcript and protein localization differ from what has been reported in *Arabidopsis.* The transcript was detected not in the stele but in the root endodermis. This was corroborated by tissue-specific RNA expression data (FACS-based tissue isolation), single-cell RNAseq, and translational reporter lines (Ortiz-Ramirez et al., 2021). Although it is unclear if this expression pattern affects protein localization, translational reporter lines showed *Zm*SHR protein is present in all cortical layers of the root (8–9 layers). This further confirms that SHR hypermobility is common in monocots. Moreover, generation of loss-of-function mutants in maize confirmed *Zm*SHR role in multilayered cortex development. Roots harboring CRISPR mutant alleles for two *Zm*SHR homologs lacked several cortex layers but maintained a functional endodermis (Ortiz-Ramirez et al., 2021). Importantly, authors demonstrated that this function was conserved in monocots by generating CRISPR mutants in *Setaria viridis*. Similar to maize, double mutants displayed a strong reduction in cortex layer number (Ortiz-Ramirez et al., 2021). Conversely, an independent study revealed that overexpression of *OsSHR2* in rice roots increases ground tissue layer number up to sixfold compared to wild type. Extra layers also had cortex identity (Henry et al., 2017).

In addition, there is evidence that *SHR* controls multilayer cortex formation in dicots as well. *Cardamine hirsuta (Ch)* is a close *Arabidopsis* relative that has been used as a model to study cortical expansion. Compared to *Arabidopsis, Cardamine* roots develop two cortex layers instead of one. *SHR* is also involved in cortex expansion in this species as heterologous expression of *ChSHR* in *Arabidopsis* produces roots with an additional cortex layer (Di Ruocco et al., 2018a). Therefore, a *SHR*-based mechanism to control cortex expansion seems to be conserved in many plant groups.

## SHR Enables Nodulation by Triggering Cortical Cell Divisions

Recently, *SHR* has been implicated in the development of root nodules, the structures that harbor the nitrogen fixing symbiont rhizobia. In *Medicago truncatula*, root nodule primordium is initiated upon rhizobia signals, which induce the dedifferentiation of cortical cells and its subsequent division (Timmers et al., 1999; Figure 1C, left). Both *SHR* and *SCR* were found to be necessary for rhizobia-induced cortex cell divisions. As shown for monocot homologs, *MtSHR* transcript and protein localization seem to be essential for cortex expansion. *SHR* expression in *Medicago* was found to be restricted to the stele, but the protein is present in the endodermis, cortex, and even the epidermis at low levels. Therefore, *Mt*SHR intercellular movement is also expanded when compared to *Arabidopsis*. Notably, SCR protein was also observed in endodermis and cortex of *Medicago* roots (Dong et al., 2021).

Importantly, cortex-specific accumulation of SHR was found to be a key aspect of nodule formation. A decrease in nodule number was observed when *MtSHR* activity was specifically repressed in cortical cells using a *SHR* dominant repressor (*MtSHR1*-SRDX). In this line, inoculation with rhizobia did not trigger as many cell divisions as in wild type roots, which prevented formation of the nodule primordium. On the other hand, cortical cell-specific *MtSHR* overexpression induced divisions even in the absence of rhizobia (Dong et al., 2021). Thus, post-embryonic cortex expansion in *Medicago* is dependent on the local activation of the *SHR/SCR* pathway. Importantly, the hormone cytokinin (6-BA) is produced in response to symbiont signals and seems to be upstream of *SHR* in this local activation pathway. Authors showed that 6-BA triggered accumulation of *Mt*SHR-GUS and *Mt*SCR, and promoted cortex divisions leading to nodule formation (Murray et al., 2007; Tirichine et al., 2007; Gauthier-Coles et al., 2018; Dong et al., 2021).

Moreover, Dong et al. (2021) demonstrated that not only the presence, but also the amount of SHR protein is crucial in triggering these divisions. As revealed by transgenic reporter lines, *Mt*SHR and *Mt*SCR proteins are present in the cortex prior rhizobia inoculation and activation of divisions. Hence, it is only when *Mt*SHR is accumulated above a threshold level, either by symbiont-produced signals, hormones, or fusion to strong cortical-specific promoters (pro*MtNRT1.3*), that cell division occurs (Figure 1C, right). Furthermore, it was found that rhizobia inoculation increases *Mt*SHR protein in cortical cells without modifying its transcript levels. This suggests that *MtSHR* post-translational regulation in a cortex-specific context (e.g., by preventing protein degradation) represents a mechanism modulating cell division in *Medicago* roots.

## A Revised Model of Root Radial Patterning

In the *Arabidopsis*-based model of root radial patterning, restriction of *SHR* movement through *SCR* interaction is a central regulatory aspect of the pathway that prevents development of more than one endodermal and one cortical cell layer. However, this function attributed to *SCR* do not seem to be conserved in other species. Both monocot and *Medicago* SCR homologs physically interact with SHR but do not limit its movement beyond the endodermis.

Alternatively, Wu et al. proposed that although *SCR* role in driving *SHR* nuclear localization is conserved, the control of SHR mobility is not. Experiments show that nuclear sequestration alone cannot explain restriction of SHR movement; thus, another factor must be involved. For example, in *Arabidopsis* roots expressing monocot homologs, ectopic expression of *AtSCR* in the stele dramatically increases *Os*SHR2 protein nuclear localization in this tissue. Because more protein is being held at the stele nuclei, a decrease in *Os*SHR2 intercellular movement should be observed. Nevertheless, no significant effect on the extent of protein mobility or decrease in cortex layer number was detected.

Another aspect of the model that requires further investigation is *SHR* role as an endodermal fate specification factor. In monocots and *Medicago,* the presence of SHR in the outer layers of the root does not result in the specification of endodermal identity. Rather, most of the GT layers where SHR protein is present have cortex identity according to their morphology and expression of genetic markers (Wu et al., 2014; Dong et al., 2021). Therefore, although *SHR* may be necessary, it is not sufficient to specify the root endodermis in these species.

A model for root radial patterning that incorporates the concept of SHR movement regulation as a central mechanism to control cortical layer number seems to be needed. In addition, work in *Medicago* suggest that local accumulation of SHR protein results in cell divisions and extra cortical layers, presumably by flipping the *SHR/SCR/CYCD6;1/RBR* circuit to its “on” state. Therefore, *SHR* can be conceptualized as a cell division trigger which can be tuned by regulating protein movement, accumulation, and degradation in a cell-type specific context. This regulation in space and time ultimately sets the asymmetric cell division program that shapes root form and function.

## The Missing Pieces

Although recent studies have enabled the identification of new regulatory aspects of the *SHR/SCR* pathway, there are important questions regarding the identity of key regulatory components (see Figure 1 question marks). For example, the identification and characterization of the factors that modulate the extent of intercellular movement is paramount. Furthermore, *SHR* is important for the specification of endodermal identity in *Arabidopsis*, but this function is not conserved in monocots and *Medicago*. Thus, identifying the developmental genetic network that specifies endodermis and cortex cell fate in other species than *Arabidopsis* will contribute to elaborate a more complete model of root radial patterning. Finally, it is unclear if SHR and SCR protein domains overlap in species with multilayered cortex, in which SHR domain is expanded to all GT layers (Wu et al., 2014; Dong et al., 2021; Ortiz-Ramirez et al., 2021). In *Medicago*, *SCR* is expressed in endodermis and cortex; hence, both transcription factors have expanded domains that overlap (Dong et al., 2021). Further analyses in monocots to determine if this expansion is conversed in complex roots will be valuable.

To achieve this, comparative high-resolution gene expression analysis and development of transgenic reporter lines in species with contrasting GT patterning is needed. Single-cell RNAseq would enable detection of differences in gene activity at the level of individual cell layers, which is essential because many developmental regulators in the root act non-autonomously. In addition, generating high-resolution gene expression networks for multiple species and comparing their architectures will contribute to map changes in genetic circuits underlying morphological diversity. Finally, SHR loss-of-function mutants in different species are needed. They will aid in dissecting SHR function in cortex development and proliferation, which is essential for harboring symbiotic associations, resist biotic and abiotic stress, and generating morphological diversity.

## Author Contributions

MH-C and CO-R wrote the article and designed the figures. All authors contributed to the article and approved the submitted version.

## Funding

MH-C and CO-R are supported by UGA-LANGEBIO Cinvestav early career funds.

## Conflict of Interest

The authors declare that the research was conducted in the absence of any commercial or financial relationships that could be construed as a potential conflict of interest.

## Publisher’s Note

All claims expressed in this article are solely those of the authors and do not necessarily represent those of their affiliated organizations, or those of the publisher, the editors and the reviewers. Any product that may be evaluated in this article, or claim that may be made by its manufacturer, is not guaranteed or endorsed by the publisher.
